# Survivorship Care for Women Living With Ovarian Cancer: Protocol for a Randomized Controlled Trial

**DOI:** 10.2196/48069

**Published:** 2024-02-09

**Authors:** Elizabeth Kvale, Farya Phillips, Samiran Ghosh, Jayanthi Lea, Claire Hoppenot, Anthony Costales, Jan Sunde, Hoda Badr, Eberechi Nwogu-Onyemkpa, Nimrah Saleem, Rikki Ward, Bijal Balasubramanian

**Affiliations:** 1 Section of Geriatrics and Palliative Medicine Department of Medicine Baylor College of Medicine Houston, TX United States; 2 Steve Hicks School of Social Work The University of Texas at Austin Austin, TX United States; 3 Department of Biostatistics and Data Science School of Public Health University of Texas Health Science Center Houston, TX United States; 4 Department of Obstetrics and Gynecology University of Texas Southwestern Medical Center Dallas, TX United States; 5 Department of Gynecologic Oncology Dan L Duncan Cancer Center Baylor College of Medicine Houston, TX United States; 6 Department of Epidemiology and Population Sciences Baylor College of Medicine Houston, TX United States; 7 University of Texas Health Houston School of Public Health - Dallas Campus Dallas, TX United States; 8 Department of Epidemiology, Human Genetics, and Environmental Sciences School of Public Health University of Texas Health Science Center Houston, TX United States

**Keywords:** chronic survivorship, metastatic survivor, metavivor, ovarian cancer, persons living with cancer, quality of life, survivor, survivorship care, survivorship transition

## Abstract

**Background:**

Ovarian cancer ranks 12th in cancer incidence among women in the United States and 5th among causes of cancer-related death. The typical treatment of ovarian cancer focuses on disease management, with little attention given to the survivorship needs of the patient. Qualitative work alludes to a gap in survivorship care; yet, evidence is lacking to support the delivery of survivorship care for individuals living with ovarian cancer. We developed the POSTCare survivorship platform with input from survivors of ovarian cancer and care partners as a means of delivering patient-centered survivorship care. This process is framed by the chronic care model and relevant behavioral theory.

**Objective:**

The overall goal of this study is to test processes of care that support quality of life (QOL) in survivorship. The specific aims are threefold: first, to test the efficacy of the POSTCare platform in supporting QOL, reducing depressive symptom burden, and reducing recurrence worry. In our second aim, we will examine factors that mediate the effect of the intervention. Our final aim focuses on understanding aspects of care platform design and delivery that may affect the potential for dissemination.

**Methods:**

We will enroll 120 survivors of ovarian cancer in a randomized controlled trial and collect data at 12 and 24 weeks. Each participant will be randomized to either the POSTCare platform or the standard of care process for survivorship. Our population will be derived from 3 clinics in Texas; each participant will have received some combination of treatment modalities; continued maintenance therapy is not exclusionary.

**Results:**

We will examine the impact of the POSTCare-O platform on QOL at 12 weeks after intervention as the primary end point. We will look at secondary outcomes, including depressive symptom burden, recurrence anxiety, and physical symptom burden. We will identify mediators important to the impact of the intervention to inform revisions of the intervention for subsequent studies. Data collection was initiated in November 2023 and will continue for approximately 2 years. We expect results from this study to be published in early 2026.

**Conclusions:**

This study will contribute to the body of survivorship science by testing a flexible platform for survivorship care delivery adapted for the specific survivorship needs of patients with ovarian cancer. The completion of this project will contribute to the growing body of science to guide survivorship care for persons living with cancer.

**Trial Registration:**

ClinicalTrials.gov NCT05752448; https://clinicaltrials.gov/study/NCT05752448

**International Registered Report Identifier (IRRID):**

PRR1-10.2196/48069

## Introduction

Ovarian cancer ranks twelfth in cancer incidence among women in the United States but fifth among causes of cancer-related death [1,2]. Treatment of ovarian cancer has benefited from recent scientific advances; however, the impact of novel treatments, including maintenance therapies, on life expectancy remains unclear [3]. Women with ovarian cancer typically complete their initial round of treatment with no evidence of disease but have a high risk of recurrence, with a majority of patients experiencing recurrence 18-24 months after the completion of initial platinum-based chemotherapy [4,5]. The focus becomes disease management, with an emphasis on treatment of cancer, minimization of toxicities, and optimization of quality of life (QOL). Historically, little attention has been paid to the survivorship needs of persons living with controlled cancer, advanced disease, and cancers that have high recurrence rates [6]. Qualitative work describes the unmet need for survivorship in this space of uncertainty, but there is little evidence to guide the delivery of survivorship care for persons living with cancer as a chronic condition.

Cancer health services science incorporated cancer survivorship as a target for care improvement following the 2006 publication of the seminal work “*From Cancer Patient to Cancer Survivor: Lost in Transition*” [7]. This foundational work summarized the challenges associated with cancer survivorship care, including the absence of systematic strategies for care provision, coordination of care across settings, unmet symptom management and psychosocial needs, and an unclear locus of responsibility for care. Subsequent years have seen dramatic increases in cancer survivorship science publications and the development of interventions and programs to meet the needs of cancer survivors. Science, however, has disproportionately focused on breast cancer and other “curable” cancers, and gaps in science and care for persons living with serious or incurable cancer remain to be addressed.

Ovarian cancer is a model of those cancers not typically encompassed in survivorship science and care. Over 19,000 women in the United States will receive a new diagnosis of ovarian cancer this year. For most of them, the point of diagnosis is the beginning of several years of living with cancer, treatment, and uncertainty. Most of these women will be diagnosed with advanced disease; 4 out of 5 patients have regional or advanced disease at the time of diagnosis. This contributes to the unfortunate outcomes associated with ovarian cancer, and 5-year survival rates remain below 50% despite improvements over the past 10 years. Women with ovarian cancer typically undergo treatments including surgery, chemotherapy, sometimes radiation therapy, and increasingly maintenance therapy with targeted therapies [8]. Survivorship care needs for women with ovarian cancer are unique, and frequently, their gynecologic oncology treatment program will also serve as the site of much of their cancer-focused survivorship care [9,10]. Most patients experience residual physical and psychological symptoms posttreatment [11]. Recurrence anxiety, psychosocial needs, sexual functioning, depressive symptoms, and uncertainty related to the care plan moving forward are all reported as sources of impaired well-being among ovarian cancer survivors [12-16]. Women with ovarian cancer being treated in safety net systems are more likely to have poor QOL and less likely to comply with follow-up visits, etc, making it more imperative to develop systems and processes to facilitate their survivorship transition.

Few studies have examined the impact of survivorship care plans (SCPs) for survivors of ovarian cancer on their QOL. The Registration System Oncological Gynecology trial was a pragmatic cluster randomized trial in which 12 hospitals were randomized to deliver computer-generated SCPs or usual care. The SCP was based on a Dutch translation of the Institute of Medicine format [7]. A total of 174 patients with ovarian cancer enrolled in the trial, of whom 61 received care at an SCP hospital and 113 received care at a usual care hospital. The primary analysis outcomes included satisfaction with care, illness perception, and health care use. There were no overall effects of SCP delivery on any of the scales of satisfaction with care; at 12 months of intention to treat (ITT) analysis, patients in the SCP arm rated the interpersonal skills of nurses lower than patients in the usual care arm. Patients in the SCP arm experienced more symptoms, were more concerned about their illness, and were more emotionally affected than patients in the usual care arm [17]. Further analyses showed increases in health care use among women with anxiety symptoms and those who received radiotherapy [18,19]. Investigators found that patients with ovarian cancer who had lower trust that the treatment would cure their disease due to the SCP reported worse emotional functioning 6 months after treatment [20]. Taken in total, the Registration System Oncological Gynecology trial underscores that for women with ovarian cancer, a templated SCP that emphasizes the frightening long-term outcomes of this disease may impair outcomes. In response to this study and the concerns of our patients, we engaged patients with ovarian cancer and providers to provide input on the development of POSTCare-O. Their input resulted in a goal related to living well with a serious illness and the inclusion of specific strategies to cope with recurrence anxiety.

We developed the POSTCare survivorship transition platform to deliver a patient-centered and Institute of Medicine (now National Academy of Medicine)–adherent SCP in breast cancer. Women with ovarian cancer experience a significant symptom burden resulting from depression and anxiety [12]. This burden is substantially higher compared with that observed in healthy populations of women and is a phenomenon that persists years into survivorship [15]. The impact of the POSTCare survivorship transition platform on reducing depressive symptom burden has been observed among survivors of breast cancer [21]. However, it remains unknown what effect the adapted intervention may have on patients with ovarian cancer. For this population, the intervention has been modified to target coaching toward the concept of “living well with a serious illness” and symptom self-management. Participants identifying a goal related to recurrence anxiety will receive a brief cognitive intervention based on acceptance and commitment therapy [22]. Strategies to address these issues in survivors of ovarian cancer are currently lacking, and this study may identify potential pathways for improved psychosocial well-being.

**Figure 1:**
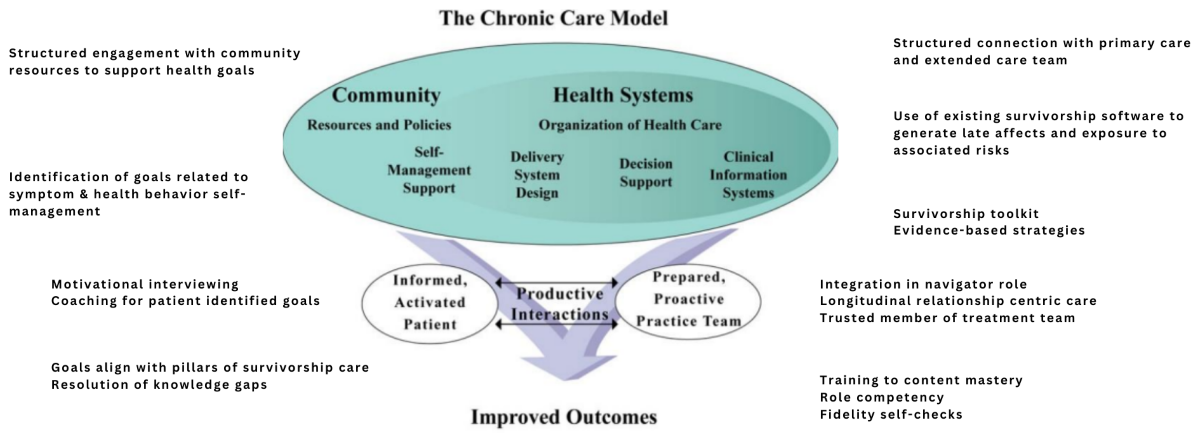
Alignment of POSTCare elements with the chronic care model. Developed by The ACT Center, formerly known as the MacColl Center for Health Care Innovation, reprinted with permission from ACP-ASIM Journals and Book.

The POSTCare survivorship care platform is framed by the chronic care model [23,24] and is also informed by the wealth of literature on care setting transition support [25-27] and patient informants (Figure 1) [28]. Designed to be delivered through telehealth or in-person, POSTCare is best understood as a health services delivery platform that coaches the survivor to engage as an activated agent in her own survivorship care. Recommendations are anchored in existing evidence-based approaches that have historically not found avenues for effective dissemination. The POSTCare platform explicitly maps onto the chronic care model essential elements, including self-management coaching and support directed at both symptoms and wellbeing for survivors. The engagement of community resources occurs with services such as exercise or mental health care to support patients’ goals. Delivery system design includes the platform’s “plug and play” approach to evidence-based behavioral change support that allows the nature of survivorship support to adjust to patients’ needs and goals. Decision support for providers delivering care is built into the platform in a “tool kit” of evidence-based behavioral interventions to support patients’ goals [29]. We hypothesize that POSTCare will increase the effective use of evidence-based care and improve outcomes for patients.

In POSTCare-O, the POSTCare platform is adapted to meet the needs of survivors of ovarian cancer. We worked with members of the CanSurvive GYN Cancer Support Group in Birmingham, Alabama, and gynecologic oncologists to identify survivorship priorities and needs. Patients with ovarian cancer and their caregivers were clear that they wanted to focus on living well during the survivorship transition, and clinicians felt that concerns that may be important for other cancer types, such as transition to primary care, might be of lesser priority in the context of ovarian cancer. They helped us understand that the essential “work” of cancer survivorship in ovarian cancer is the work of living well despite serious illness and the specter of mortality. Using the palliative dual framework, a technique used to assist patients in the task of living well despite serious illness [30,31], and a brief acceptance and commitment therapy intervention [22], we have incorporated a goal focused on living well with a serious illness that was not a component of the breast cancer POSTCare platform.

The overarching goal of this study is to test processes of care that support outcomes, including QOL, in survivorship. We will use QOL as a primary outcome, but we will also look at factors such as recurrence worry, depressive symptom burden, and survivorship efficacy that may also be influenced by improved care processes. The specific aims of this study are to conduct a randomized controlled trial (RCT) enrolling 120 women with advanced ovarian cancer. We will test the efficacy of the POSTCare platform in supporting QOL, reducing depressive symptom burden, and reducing recurrence worry. In our second aim, we will examine factors associated with the impact of the intervention. Our final aim focuses on understanding aspects of care platform design and delivery that are likely to affect the potential for dissemination. We will use both qualitative and quantitative methods to assess patient experience, provider experience, and pragmatic aspects of clinical implementation, with the goal of redesigning the implementation for greater dissemination potential.

## Methods

### Study Design

**Figure 2:**
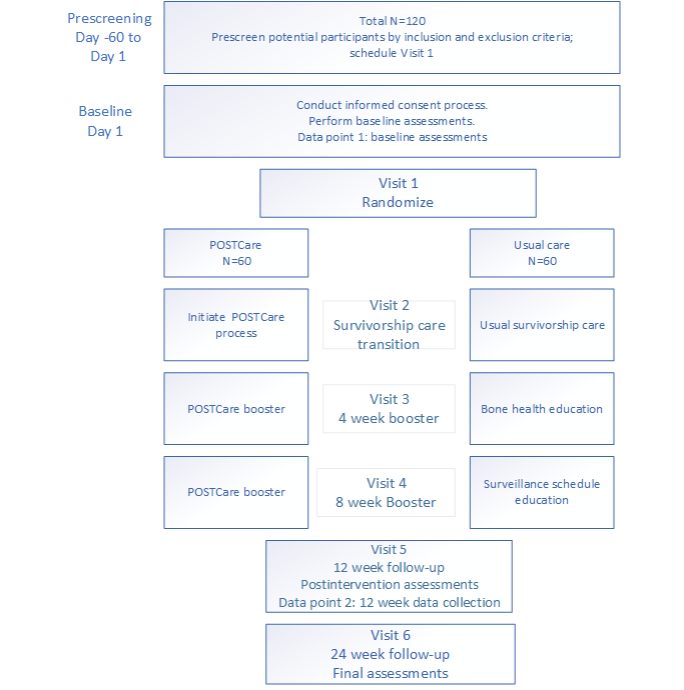
POSTCare-O randomized clinical trial flow demonstrating enrollment, randomization, and data collection time points.

**Figure 3:**
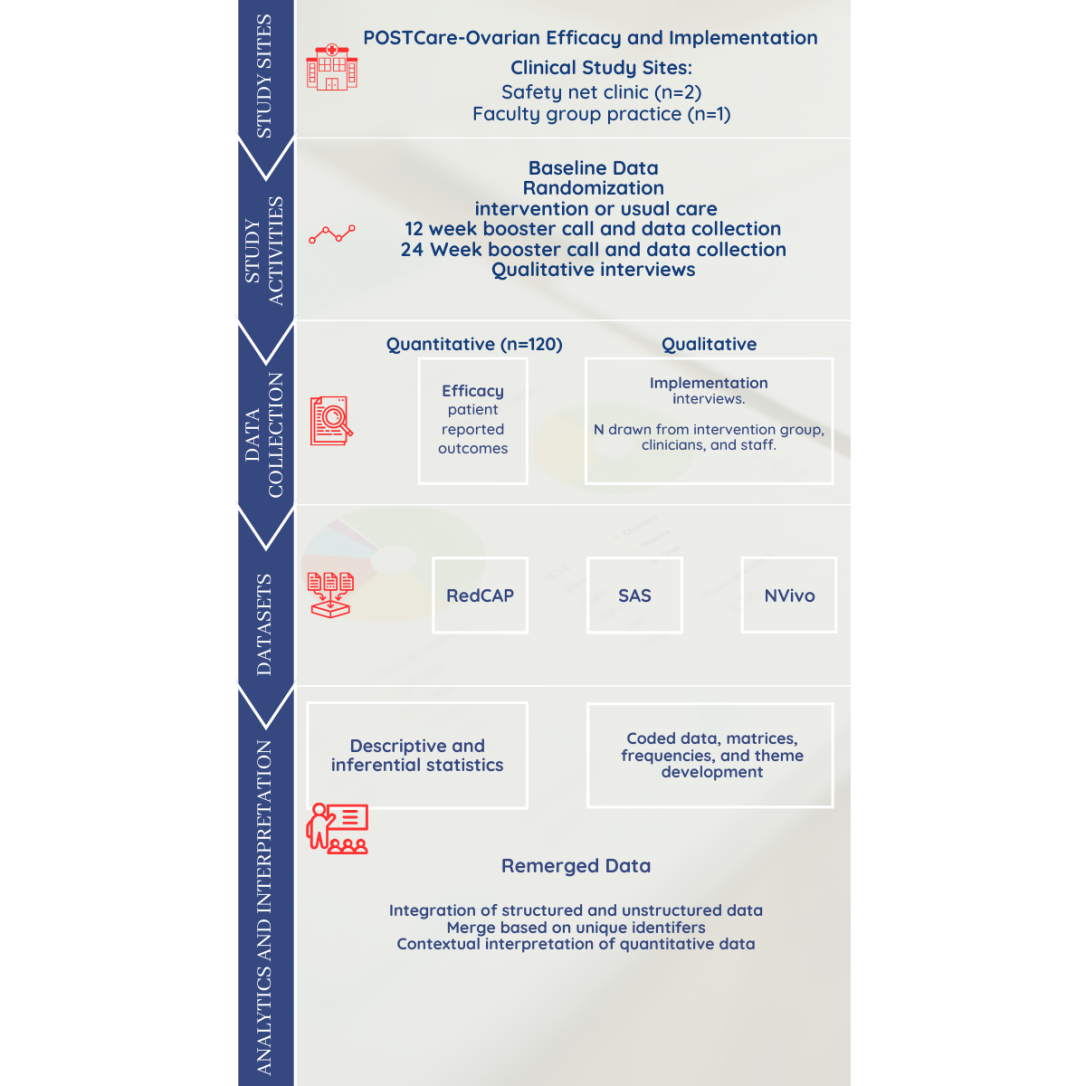
Mixed methods framework demonstrating data sources and integration schema.

We will conduct a 2-arm RCT to evaluate the impact of a telehealth-delivered survivorship transition care platform. Survivors of ovarian cancer (N=120) will be randomly allocated to receive survivorship care either using the POSTCare Platform or standard of care. Study design and reporting will be in accordance with the CONSORT (Consolidated Standards of Reporting Trials) checklist. We will use quantitative and qualitative methodologies in a concurrent triangulation mixed methods design using qualitative data to augment our interpretation of quantitative data. Outcomes will be collected at baseline, 12 weeks, and 24 weeks, with the primary outcome being a QOL assessment at 12 weeks after the survivorship transition (Figures 2 and 3).

### Sample Size

We will enroll 120 women completing primary treatment for stage 2-4 ovarian cancer from 3 urban gynecologic oncology clinics located in the US state of Texas. Participants will have received some combination of surgery, chemotherapy, radiation therapy, and biologics. Continued maintenance therapy is not an exclusion factor. The disease-stage sample frame was developed with input from our gynecologic oncology collaborators based on treatment exposures and the similarity of survivorship challenges. Aim 1 proposes to implement a RCT devised to compare QOL measures among patients with ovarian cancer randomized to receive usual care versus the POSTCare survivorship care transition program. The Functional Assessment of Cancer Therapy-Ovarian (FACT-O) QOL survey will be collected at baseline as well as 12 and 24 weeks after the initial course of adjuvant chemotherapy. The primary end point will be the 12-week survey. The sample size of 120 patients provides at least 80% power to detect a 7% increase in the mean FACT-O score for women randomized to the POSTCare survivorship care intervention. This is sufficient to ascertain a minimally important difference of 8 points [32].

### Recruitment and Setting

We will recruit participants from gynecologic oncology practices at 3 clinic settings in Texas: 1 safety net practice located in Dallas, 1 safety net practice located in Houston, and 1 faculty group practice located in Houston. Cumulatively, the sites serve approximately 140 eligible patients per year and ensure a diverse population of 120 participants can be recruited during the 24-month recruitment period. A total of 60 participants will be randomly assigned to the intervention group and receive care using the POSTCare process, and another 60 will be randomly assigned to the control group. To be included, patients must (1) be diagnosed with ovarian cancer at the age of 18 years or older; (2) be within 3 months of completing initial treatment for stage 2-4 ovarian cancer. Treatment may include surgery, chemotherapy, radiation therapy, immunotherapy, or other biologics. Participants may be on maintenance therapy; and (3) be able to provide consent in English or Spanish. Patients who are enrolled in hospice care directly following the treatment conclusion will not be eligible for the study. It is anticipated that this study sample will reflect the ethnic and racial diversity of our clinical settings.

### Recruitment Procedures

Study research coordinators will have access to the electronic medical record at each site. They will collaborate with clinical personnel to identify patients who will be completing initial therapy. Coordinators will work to identify patients in treatment at least 8 weeks before the completion of therapy. They will identify an upcoming clinical appointment, either in clinic or infusion, where the team will approach patients to provide information about the study, answer questions they may have, and obtain informed consent. Enrolled participants will be asked to complete a web-based, self-administered survey using a smart device to collect baseline measurements before randomization.

### Randomization

After the baseline survey is administered, the biostatistician will use SAS (SAS Institute) to perform a permuted stratified blocked randomization [33]. The stratification variable used maintenance therapy versus no maintenance therapy. The rationale for this stratification is that those patients with ovarian cancer who receive maintenance therapy with a poly (ADP-ribose) polymerase (PARP) inhibitor may experience a different symptom burden or illness trajectory that impacts outcomes [3]. Survivors will be randomly allocated to either the control group or the POSTCare intervention group with a 1:1 ratio (N=120). Stratification will be based on disease stage and maintenance therapy (a PARP inhibitor vs Bevicizumab vs no maintenance). Each of these factors is associated with overall QOL and symptom burden. Participants, clinicians, data collectors, biostatisticians, and investigators will be blinded to allocation. The research nurse interventionist (described below) will not be blinded but will not conduct study activities related to measuring, collecting, or interpreting outcomes. Although this is a low-risk intervention, unblinding decisions will be made by the investigators in the context of serious adverse events attributable to the study. If a decision is made to unblind a participant, the study data coordinating center will undertake the unblinding and protect the confidentiality of treatment assignments for other participants. The treatment assignment will be communicated to the participant and the participant’s treating physician. The unblinding event will be reported to the Baylor College of Medicine institutional review board, documented in trial records, and the analysis of study outcomes may be adjusted to account for the unblinded participant to ensure that the integrity of the overall trial results is maintained. Criteria for study dropout include withdrawal of consent, failure to adhere to protocol, loss to follow-up, health changes making further participation burdensome, and personal reasons or changes in life circumstances.

### Control Group

The control group will receive standard survivorship care delivered through telehealth. All sites have a standard survivorship visit that includes the use of a software package to generate the delivery of a paper SCP based on American Society of Clinical Oncology guidelines. This comprises a treatment summary, an upcoming surveillance visit schedule, and guidance on late effects. This visit includes the delivery of information and specifically does not include goal setting, use of the dual framework technique (described in the “Intervention” section), acceptance and commitment intervention (described in the “Intervention” section), self-management coaching, or the use of motivational interviewing techniques. To ensure fidelity to control group standards, all visits will be audio-recorded and reviewed to evaluate for evidence of contamination of the control condition. Telephone-based booster contacts will serve as attention control with structured delivery of educational material on surveillance visits and bone health.

### Intervention

POSTCare is a structured cancer survivorship navigation platform that seeks to improve cancer outcomes for survivors. Key elements of the platform include personalized self-management support and tailored SCP delivery. The platform is delivered through telehealth (a video platform) and comprises an initial survivorship care transition visit that includes the development and delivery of a personalized care plan. Within the platform framework, a trained nurse uses motivational interviewing and communication skills to engage patients in the development of a POSTCare plan that incorporates health goals and strategies related to surveillance, symptom management, and health behavior [34,35]. Survivor engagement with the POSTCare plan is supported by monthly navigator phone booster follow-up for 2 months, also delivered by the nurse survivorship navigator. Participants will be offered an additional nonstructured phone check-in before their first follow-up visit at 3 months.

### Patient-Centered Design

The POSTCare session begins with the coach engaging the survivor in sharing her cancer treatment narrative, anchoring the activity to the patient’s experience and needs. The identification of health goals is the central activity of POSTCare and distinguishes this approach to survivorship care from those that simply deliver information. The survivor is asked to think about identifying 1 or more goals in the following survivorship domains: social support, healthy habits, symptom management, and coping with uncertainty. Resource and activity support materials related to goals are maintained in the POSTCare-O web-based survivorship toolkit and used by coaches. As an adaptation for the needs of survivors of ovarian cancer, the dual framework [31] is introduced by coaches in goal-setting as a strategy for living with uncertainty. The dual framework is a strategy used in palliative encounters to help patients focus on what living well means to them while holding the possibility of advancing illness or death in the same cognitive frame. It provides a structure to anchor the focus on living well across the longitudinal trajectory of survivorship navigation. We will introduce a brief cognitive exercise derived from acceptance and commitment therapy to address recurrence worry if participants articulate this as a survivorship concern and goal [22,36-38]. Our informants indicated that using a focus on what it means to live well despite serious illness is an acceptable palliative intervention even at the initiation of the survivorship period when they wish to be focused on the positive aspects of treatment completion.

The coach and survivor strategize about potential barriers to goal accomplishment and explore ways to address barriers, with the coach using motivational interviewing techniques to explore survivor ambiguity about health goals and nurture self-efficacy in working toward goals. The components of the platform, drawn from an evidence-based approach to care setting transition [24,25,27,39-41], include an emphasis on survivor engagement and activation. The average length of the coaching session is 75 minutes, which includes the creation of the SCP [21].

Booster survivorship navigation telephone contacts occur at 4 weeks and 8 weeks after baseline. The survivorship coaches will review health goals, including living well, adjust goals as needed, discuss progress, identify barriers, and brainstorm about strategies to overcome barriers. The survivorship coaching intervention is delivered through telehealth by the nurse survivorship navigation coach. For participants who do not have a smart device, the intervention can be delivered through a smart device provided by the study in a clinic setting.

### Qualitative Methods

Qualitative methods will be used to inform the adaptation and refinement of the intervention. Interviews will be undertaken with 3 groups of informants: women living with ovarian cancer; nurses who have been trained in the POSTCare model; and gynecologic oncologists, clinic staff, and administrators. Semistructured exit interviews will be conducted by specially trained research team members within 30 days of completing the intervention. Although the exit interview questions will be stated as broad questions, the researcher will be trained to probe for details, including asking for specifics and operational examples.

We will partner with women enrolled in this study to conduct semistructured interviews on the POSTCare survivorship care experience. Interview topics will include (1) timing of survivorship care—both initiation of survivorship transition and longitudinal care; (2) critique of proposed intervention content and existing materials; (3) understanding the meaning of this intervention for participants; (4) exploring individual differences between experiences and outcomes; and (5) evaluation of intervention length, intensity, frequency, and mode of delivery. Interviews will be conducted with gynecologic oncology nurses who have completed the POSTCare-O web-based training and used components of the POSTCare model to provide the intervention. Finally, we will explore the same interview topics with clinic staff at each study site to learn what elements of the POSTCare model work well in their clinical setting, which elements they are able to use routinely, which components seem to most meet the needs of their patients, and which elements of survivorship care they find most satisfying.

### Outcome Measures

We will use the FACT-O instrument as the primary end point for the clinical trial. The FACT-O is a 38-item assessment that comprises a core QOL instrument (the FACT-General) and a 12-item ovarian module. The internal consistency for the complete instrument is Cronbach α=.92, and the test-retest reliability is *r*=0.81 [42]. The subscales demonstrate similar acceptable psychometrics. This instrument is widely used in trials and will allow us to meaningfully compare the results of this trial to those of other studies that examine QOL. The minimally important difference is the “smallest difference” in FACT-O scores that patients perceive as clinically important and is 3-8 points [43]. We have powered the study to identify this level of change.

The Patient Health Questionnaire-9 (PHQ-9) is a widely used screening tool for depression, consisting of 9 items [44]. It takes approximately 5 minutes to complete and has been found to be a valid and reliable measure of depressive symptoms in various populations, including patients with cancer. The total score on the PHQ-9 can range from 9 to 27, with higher scores indicating a higher level of depressive symptom burden. A study by Thekkumpurath et al [45] found that the PHQ-9 was a valid and reliable measure of depression in patients with cancer and recommended its use in clinical practice. Additionally, the PHQ-9 has been used as an outcome measure in various interventions for depression, including those targeting patients with cancer [46]. The PHQ-9 has demonstrated good internal consistency and test-retest reliability, with a coefficient α=.89 and an intraclass correlation coefficient of 0.84, respectively [44].

The Fear of Cancer Recurrence-7 (FCR-7) is a 7-item questionnaire designed to offer a psychometrically sound assessment of fear of cancer recurrence with a limited response burden. The instrument comprises 7 questions: 5 that use a 5-point Likert scale ranging from 1 (not at all) to 5 (all the time), and a single question that uses an 11-point Likert scale ranging from 0 (not at all) to 10 (a great deal). Total scores on the measure range from 6 to 45 [47]. A cutoff score of 17 or above reflects moderate fear of cancer recurrence, and a score of 27 or above indicates severe fear of cancer recurrence. The measure demonstrates good internal consistency (Cronbach α=.92) and validity as compared to measures of anxiety and depression [48].

Other measures used as secondary outcomes and potential predictors were selected with a priority on acceptable psychometric performance in similar populations and acceptable performance in one of our previous studies.

“Aim 1” proposes to implement a RCT devised to compare QOL measures among patients with ovarian cancer randomized to receive usual care versus the POSTCare survivorship care transition program. Outcome measures, including the FACT-O QOL survey, the FCR-7 survey, and the PHQ-9, will be collected at baseline as well as 12 and 24 weeks after the initial course of adjuvant chemotherapy. The primary end point will consist of the 12-week FACT-O survey. The sample size of 120 patients provides at least 80% power to detect a 7% increase in the mean FACT-O score for women randomized to the POSTCare survivorship care intervention. This is sufficient to ascertain a minimally important difference of 8 points. Secondary outcomes (Table 1) will also be assessed at 12 and 24 weeks. Baseline descriptive statistics will be presented by site. Longitudinal analysis of the FACT-O QOL scores will use a mixed effects linear model with a restricted maximum likelihood estimation method, and an unstructured covariance matrix will be used to estimate trends. An interaction between intervention and time will be estimated to explore the effectiveness of POSTCare for time-varying trends [49]. Though the mixed effect model can accommodate some degree of missing data under the ignorability assumption, we also plan to use multiple imputations using the random forest method [50,51] to accommodate missing data (if greater than 10% of survey items), which will be assumed to be missing at random. In the event of a sign of a violation of that assumption, a pattern mixture model will be used to mitigate the effect of informative missingness. Secondary analyses will evaluate relationships between baseline and 12-week FACT-O scores and clinical end points progression-free survival (PFS) and overall survival (OS). Cox proportional hazard models will be used to estimate the relative risk of PFS and death per increase in FACT-O [52]. Ties in failure times will be handled with the approximate likelihood of Efron. PFS and OS will be measured from the date of randomization. PFS is defined as the minimum amount of time until clinical progression, death, or the date of last contact. OS is the duration from randomization to death or to the date of last contact (right-censoring). As is customary, secondary analyses are not powered but may help generate additional evidence that needs to be tested in the future by a properly powered study.

**Table 1:** Summary of variables and outcomes.

Variables for outcome analysis			Measures	Reliability, *r*	Source
**Primary outcomes**					
		Quality of Life	Functional Assessment of Cancer Therapy-Ovarian [42]	.92	Patient self-report
		Depression	Patient Health Questionnaire-9 [45]	.94	Patient self-report
		Recurrence anxiety	Fear of Cancer Recurrence-7 [47]	.93	Patient self-report
**Secondary outcomes**					
		Patient self-efficacy	Stanford Chronic Illness Self-Efficacy Scale [53] Patient Activation Measure [54]	.91 .87-.91	Patient self-report
		Satisfaction with communication	Stanford communication with physicians [55]	.89	Patient self-report
		Health care use	Stanford Health Care Utilization [53]	.76-.97	Patient self-report
		Satisfaction with care coordination	Stanford Self Efficacy [55]	.91	Patient self-report
		Perception of Informational Support	Patient-Reported Outcomes Measurement Information System: informational support measure [56]	—^a^	Patient self-report
		Symptom Burden	MD Anderson Symptom Inventory for ovarian cancer Stanford Social/Role Activities Limitations	.89-.90 .91	Patient self-report
**Predictor variables**					
	Demographic or medical information		Demographic questionnaire: will include gender, race, and treatment history	N/A^b^	Electronic medical record
	Cancer coping style		Brief version of the Coping Orientation to Problems Experienced [57]	.75	Patient self-report
	Social support		Social Provisions Scale [58]	.92	Patient self-report
	Education level		Stanford Education [59]	N/A	Patient self-report

^a^Not available.

^b^N/A: not applicable.

“Aim 2” applies mediation analysis to the data acquired from the RCT proposed in Aim 1. Aim 2 explores the potential for heterogeneity in the effectiveness of the POSTCare survivorship program. The primary end point for aim 2 is QOL at 6 months, as measured by the FACT-O. Combining both study arms, subgroup analysis will assess the distributions of potential prognostic factors for QOL at 6 months. Analyses will adjust for statistically significant prognostic factors. Mediation modeling will be applied to decompose the relationships among the care plans, intermediate surrogate markers of QOL acquired during the course of follow-up, and QOL at 6 months. Surrogate markers include patient activation, goal setting, self-efficacy, and care satisfaction, to be collected at 12 weeks and 24 weeks from surveys identified in Table 1. For each surrogate marker, an intermediate response will be defined. The direct and indirect impact of POSTCare will be estimated for each surrogate response using mediation analysis [60-62] to elucidate the causal mechanisms of QOL and assist with planning for larger confirmatory studies and studies in other disease types.

Qualitative data for intervention adaptation will be analyzed using a thematic analysis framework. At the outset of the process of analysis and interpretation, the qualitative team will read each transcript from interviews in its entirety to achieve a global sense of substance and context. Working independently of one another, we will engage in a line-by-line search for recurring ideas, coding each transcript for themes. After identifying dominant themes, we will evaluate the degree of consensus among participants. An initial codebook will be developed from the interview guide and adaptation framework. A master code book will be entered into NVivo (version 11; QSR International). The data will be merged with the quantitative data to inform interpretation and draw stronger inferences. As described by Farquhar et al [63], mixed methods are particularly beneficial where the interventions are complex and the platform for evaluation and identification of suitable outcomes is challenging. Insights gained from quantitative and qualitative approaches complement each other to provide a more in-depth understanding. This deeper understanding can inform the process of refining interventions and hypothesis generation and facilitate replication of the intervention through greater knowledge of the active components and potential barriers to implementation. Moreover, qualitative research can be used to examine and address key uncertainties before dissemination efforts [64]. Interviews will be conducted by experienced qualitative interviewers on our established research team. We anticipate conducting up to 30 interviews at the outset of the process of analysis and interpretation*.* We will use all data sources to inform the revision of the POSTCare-O platform before active dissemination [65-68].

### Ethical Considerations

Ethical approval for this trial has been obtained through the Baylor College of Medicine Institutional Review Board (H52939). All participants in the trial will provide informed consent before the initiation of study activities. The primary risk in this study is a risk to confidentiality. All study data will be uploaded directly to the data coordinating center, REDCap (Research Electronic Data Capture; Vanderbilt University), which is implemented to be compliant with HIPAA (Health Insurance Portability and Accountability Act) standards. Participants will receive compensation for their participation in the trial in a longitudinal manner, with US $10 at baseline and 12 week data collection timepoints and US $30 at the 24 week time point. Total participant compensation is US $50.

## Results

This study will be conducted over a period of 3 years. Data collection was initiated in November 2023 and will continue for approximately 2 years. Approval for the study protocol has been obtained from the institutional review board of Baylor College of Medicine, and a reliance agreement has been approved by the University of Texas Southwestern Medical Center and Parkland Health. We will report on the outcomes identified above as a primary study activity. We also plan to collaborate with patients and clinicians to identify adaptations to the POSTCare-O platform to optimize dissemination potential. Results from this study will inform preparation to study survivorship care for patients living with advanced cancer and other disease types. We expect results from this study to be published in early 2026.

## Discussion

### Overview

The overarching goal of this research program is to improve QOL and well-being for women living with ovarian cancer. This paper details the protocol for a randomized, controlled, dual-blinded study testing a survivorship care platform for women completing initial treatment for ovarian cancer. The gaps in science related to the care of patients with advanced or incurable cancer are thoughtfully articulated in a recent National Cancer Institute meeting report [6].

This study will address several of the gaps in science that exist relative to women living with ovarian cancer. Much of the science focused on the needs of survivors of cancer has been conducted in breast cancer populations; however, a solid body of work exists that characterizes the impact of ovarian cancer and treatment on QOL, sexual well-being, care preferences, and health care use. This study, with a collection of measures related to symptom burden and psychosocial well-being, will characterize unmet needs at baseline among survivors of ovarian cancer, and we will be able to place our work in the context of the existing science for interpretation. Further, our qualitative stream will provide additional context for interpretation of our quantitative data as we work to better understand the patients’ experience.

Although overt efforts have been made to minimize limitations by design, this study will have several limitations. While we expect to meet our sample size target of recruiting 120 women with ovarian cancer, practical aspects of participant recruitment and budgetary constraints will constrain our ability to stratify the population and evaluate potential mediators. Furthermore, the highly structured nature of care delivery for this study, including consistent intervention delivery by a single research nurse, may not reflect the conditions encountered in a “real-world” implementation of this health services intervention that is intended to be optimized for dissemination. While we have put in place measures to address concerns related to intervention fidelity, including review of recordings of intervention sessions with fidelity checklists and ongoing education to ensure consistent delivery, inconsistent delivery remains a potential source of bias for this study. This study will take place at 2 US “safety net” clinics that serve economically disadvantaged patients primarily, as well as a university practice that serves individuals with private or federal insurance. While this design should give us a representative sample within our geographic region, the results may not be generalizable to other regions of the United States or health care systems outside the United States. Despite these limitations, this study will make a significant contribution to survivorship science in ovarian cancer by examining aspects of supportive care that may improve QOL for patients.

## Abbreviations

CONSORT: Consolidated Standards of Reporting Trials
FACT-O: Functional Assessment of Cancer Therapy-Ovarian
FCR-7: Fear of Cancer Recurrence-7
HIPAA: Health Insurance Portability and Accountability Act
ITT: intention to treat
OS: overall survival
PARP: poly (ADP-ribose) polymerase
PFS: progression-free survival
PHQ-9: Patient Health Questionnaire-9
QOL: quality of life
RCT: randomized controlled trial
REDCap: Research Electronic Data Capture
SCP: survivorship care plan

## Data Availability

The data sets generated during and/or analyzed during this study are available in the Palliative Care Research Collaborative De-Identified Data Repository [69].
